# Osteoactivin/GPNMB as a candidate biomarker of diabetic nephropathy in type 2 diabetes: associations with angiogenic and kidney injury biomarkers

**DOI:** 10.3389/fendo.2026.1908607

**Published:** 2026-08-06

**Authors:** Mohamed Abu-Farha, Sulaiman Marafie, Laila Alhemoud, Eman Al Shawaf, Reem Malek, Joud Almuharib, Sriraman Devarajan, Irina Al-Khairi, Preethi Cherian, Hamad Ali, Abdullah Faisal Almutairi, Ibrahim Taher, Mohammad Shehab, Fahad Al-Ajmi, Fahd Al-Mulla, Abdulnabi Al Attar, Jehad Abubaker

**Affiliations:** 1 Department of Biochemistry and Molecular Biology, Dasman Diabetes Institute, Kuwait City, Kuwait; 2 Department of Translational Research, Dasman Diabetes Institute, Kuwait City, Kuwait; 3 National Dasman Diabetes Biobank, Dasman Diabetes Institute, Kuwait City, Kuwait; 4 Department of Medical Laboratory Sciences, Faculty of Allied Health Sciences, Health Sciences Center, Kuwait University, Kuwait City, Kuwait; 5 Family Medicine, King Saud Medical City, Riyadh, Saudi Arabia; 6 Microbiology Unit, Department of Pathology, College of Medicine, Jouf University, Sakaka, Saudi Arabia; 7 Medical Department, Amiri Hospital, Ministry of Health, Kuwait City, Kuwait

**Keywords:** ANGPTL8, diabetic nephropathy, GPNMB, osteoactivin, type 2 diabetes

## Abstract

**Background:**

Diabetic nephropathy (DN) is a main complication of type 2 diabetes (T2D) and a major cause of chronic kidney disease and end stage renal failure. Early detection remains challenging, which has attracted interest in exploring novel biomarkers. Osteoactivin is a protein involved in inflammation, tissue repair, and metabolic stress, but its role in DN is not yet fully understood.

**Objective:**

To evaluate circulating osteoactivin as a potential biomarker of DN, and to examine its relationship with renal dysfunction, angiogenic markers, and diagnostic performance.

**Methods:**

This cross-sectional study included 42 non-diabetic controls, 50 patients with T2D, and 67 patients with DN. Plasma osteoactivin levels were measured using a customized Luminex multiplex assay. Group differences were analyzed using one-way ANOVA or the Kruskal– Walli’s test, with *post hoc* comparisons performed where appropriate. Spearman’s correlation was used to assess associations between osteoactivin and various nephropathy-related biomarkers, including Angiopoietin-1, Angiopoietin-2, ANGPTL3, ANGPTL4, ANGPTL6 and ANGPTL8. Multinomial logistic regression was used to examine associations with disease groups. Receiver operating characteristic (ROC) curve analysis was performed to assess diagnostic performance.

**Results:**

Circulating osteoactivin levels increased progressively from controls to T2D and were highest in DN (P < 0.001). Higher osteoactivin levels were associated with worse kidney function and showed positive associations with selected angiogenic markers, particularly Angiopoietin-2 (r = 0.414, P < 0.001), ANGPTL4 (r = 0.406, P < 0.001), and ANGPTL8 (r = 0.165, P = 0.039). ROC analysis showed that osteoactivin alone had moderate diagnostic performance (AUC = 0.786), but this improved substantially when combined with angiogenic markers, especially ANGPTL8 (AUC = 0.881), supporting the added value of a multi-marker approach.

**Conclusion:**

Circulating osteoactivin is associated with renal dysfunction and angiogenic activity in DN and demonstrated moderate diagnostic performance. This was further enhanced when combined with other angiogenic biomarkers, particularly ANGPTL8. These findings support its potential as a non-invasive biomarker tool for the detection and assessment of disease severity in DN.

## 1 Introduction

Type 2 diabetes (T2D) is a chronic metabolic disease defined by sustained hyperglycemia due to impaired insulin secretion, reduced peripheral insulin action, or a combination of both (1, 2). It is accompanied by the progressive dysfunction of the pancreatic beta cells affecting insulin secretion, which in turn, further contributes to the disruption of glycemic control (3, 4). According to the International Diabetes Federation (IDF) Diabetes Atlas, an estimated 589 million adults worldwide were living with diabetes in 2024, highlighting the major global burden of disease (5). This burden is especially relevant to Kuwait where diabetes affected an estimated 908,500 adults in the same year, corresponding to a prevalence of 25.6% (6). T2D is associated with a wide range of chronic complications affecting overall morbidity and/or mortality. Such complications are classified into either macrovascular or microvascular complications. Macrovascular complications involve damage to large blood vessels that include cardiovascular disease, cerebrovascular disease, and peripheral arterial disease. On the other hand, microvascular complications are caused by damage to small blood vessels and primarily include diabetic retinopathy, neuropathy, and nephropathy (1, 3).

Diabetic nephropathy (DN) is another example of a major microvascular complication of diabetes affecting up to 40% of individuals with diabetes (3, 7, 8). It is caused by a series of cellular dysfunctions like prolonged hyperglycemia, inflammation, oxidative stress, and hypertension, which together lead to progressive renal injury. Hyperglycemia promotes oxidative damage and the formation of advanced glycation end products, whereas inflammatory and hemodynamic processes further contribute to endothelial dysfunction and structural damage within the renal microvasculature and glomeruli (7, 9). DN is characterized by changes in albuminuria levels and reduced kidney function, both of which are defined by albumin-to-creatinine ratio (ACR) greater than 30 mg/g and glomerular filtration rate (eGFR) of less than 60 mL/min/1.73 m², respectively (7, 8). This has led to growing interest in identifying biomarkers that could potentially improve the early detection, monitoring, and risk stratification of DN.

Osteoactivin, also known as glycoprotein non-metastatic melanoma protein B (GPNMB), is an endogenous type I transmembrane glycoprotein expressed in a wide range of cell types, including osteoblasts, osteoclasts, melanocytes, keratinocytes, microglia, macrophages, and dendritic cells (4, 10). It plays a key role in several biological processes including bone remodeling, tissue repair, inflammation, and cellular differentiation. Beyond its physiological roles, increased expression of osteoactivin has been reported in a variety of inflammatory and chronic diseases such as renal disease, cancer, neurodegenerative disorders, atherosclerosis, and myocardial injury. This suggests its potential involvement in key cellular pathways related to tissue injury, inflammation, and vascular dysfunction (4, 7). Moreover, emerging evidence further suggests osteoactivin may also play a role in endothelial function and vascular homeostasis, where its soluble extracellular domain has been reported to promote angiogenesis and endothelial cell recruitment (10, 11). However, the precise mechanism of vascular regulation is not fully understood.

Taken together, these studies along with its reported elevated levels in T2D, circulating osteoactivin could highlight the vascular and renal injury pathways involved in DN, supporting its potential role as a non-invasive biomarker for early disease detection and management. This study investigated circulating osteoactivin levels in individuals with T2D and DN to explore its potential role as a biomarker of renal injury and angiogenic dysregulation. We further examined its relationships with established nephropathy-associated angiogenic markers, including Angiopoietin-1, Angiopoietin-2 (12, 13), ANGPTL3 (14), ANGPTL4 (15), ANGPTL6, and ANGPTL8 (16), to better characterize its involvement in the pathophysiology of DN. In addition, receiver operating characteristic (ROC) curve analysis was performed to evaluate the ability of circulating osteoactivin to discriminate individuals with DN, thereby assessing its potential diagnostic utility as a biomarker of disease progression.

## 2 Materials and methods

### 2.1 Study design and population

This study was a cross-sectional observational study where a total of 159 adults were included and classified into three groups; non-diabetic control group (n = 42), T2D group without nephropathy (n = 50), and DN group (n = 67). Clinical, biochemical, and renal function parameters were assessed in all participants, and circulating osteoactivin together with selected angiogenic markers were measured in plasma samples.

Participants were recruited and enrolled in the study after providing written informed consent at Dasman Diabetes Institute in Kuwait. The study protocol was approved by the Ethical Review Board of Dasman Diabetes Institute and was conducted in accordance with the ethical principles of the Declaration of Helsinki.

### 2.2 Study group definitions

Participants were categorized into three groups according to their glycemic and renal status. The first group included individuals without diabetes with normal renal function. The second group included participants who had a clinical diagnosis of T2D but showed no evidence of DN. The third group included individuals with T2D and evidence of DN defined by the presence of albuminuria indicated by ACR greater than 30 mg/g, and impaired renal function reflected by eGFR of less than 60 mL/min/1.73 m².

### 2.3 Quantification of osteoactivin and angiogenic markers

Fasting venous blood samples were collected from all participants into ethylenediaminetetraacetic acid (EDTA) anticoagulant tubes. Samples were processed promptly by centrifugation to separate plasma from cellular components. The resulting plasma was carefully aliquoted into multiple labeled microtubes (or assay plates) to avoid repeated freeze–thaw cycles and stored at −80 °C until further analysis.

Before analysis, plasma aliquots were thawed on ice and diluted using the sample diluent supplied with the assay kit according to the manufacturer’s instructions (dilution factor 1:2) for osteoactivin, Angiopoietin-1, Angiopoietin-2, ANGPTL3, ANGPTL4, ANGPTL6. Circulating concentrations of osteoactivin and selected angiogenic markers were quantified using a customized magnetic bead-based multiplex assay (Magnetic Luminex^®^ Premixed Multi-Analyte Kit, Cat. #LXSAHM, R&D Systems, Minneapolis, MN, USA). The assay was performed according to the manufacturer’s recommended protocol. Briefly, standards, quality controls, and diluted plasma samples were incubated with antibody-coated magnetic microspheres, followed by sequential incubation with biotinylated detection antibodies and streptavidin-phycoerythrin. Following the required washing steps, fluorescence intensity was acquired using a Luminex^®^ detection system Bioplex 200 (Bio-Rad, CA, US), and analyte concentrations were calculated from standard calibration curves generated using the manufacturer’s analysis software Bioplex Manager (Bio-Rad, CA, US).

Strict quality control measures were implemented throughout the analytical procedure. All plasma samples were thawed only once and maintained on ice during assay preparation to preserve analyte integrity. Repeated freeze–thaw cycles were avoided to minimize protein degradation and analytical variability. Samples, standards, and quality controls were analyzed in accordance with the manufacturer’s recommendations to ensure consistency and reproducibility.

The assay demonstrated excellent analytical performance, with intra-assay and inter-assay coefficients of variation ranging between 5% and 10% for all analytes measured. The lower limit of detection was 0.75ng/mL for osteoactivin, Angiopoietin-1and Angiopoietin-2 (lower detection limit was 0.1 ng/mL), ANGPTL3 and 6 (Lower detection limit was 0.3ng/mL) and ANGPTL4 (lower detection limit was 1.5ng/mL). No significant cross-reactivity or assay interference was observed among the analytes included in the multiplex panel. Assay accuracy was confirmed by comparing experimentally measured standard concentrations with their theoretical values, which consistently fell within the accepted recovery range of 95–100%, indicating high assay precision and reliability.

Circulating ANGPTL8 concentrations were measured using a commercially available enzyme-linked immunosorbent assay (ELISA) kit (Eiaab Science, Wuhan, China; Catalogue No. E11644h), according to the manufacturer’s instructions. Plasma samples were thawed on ice and diluted 1:10 using the sample diluent provided with the kit. Standards were prepared according to the manufacturer’s protocol and loaded onto the ELISA plate alongside the diluted plasma samples.

Following completion of the assay procedure, optical density (OD) was measured using a Synergy™ H4 Multi-Mode Microplate Reader (Bio Tek, Santa Clara, CA, USA). A standard calibration curve was generated by plotting the known concentrations of the standards against their corresponding OD values, and the concentrations of the unknown samples were calculated from the resulting standard curve equation.

All samples were analyzed in accordance with the manufacturer’s recommendations, and appropriate quality control procedures were followed throughout the assay. ELISA demonstrated excellent analytical performance, with an intra-assay coefficient of variation (CV) ranging from 2.0% to 5.0% and an inter-assay CV of less than 10%, indicating high precision and reproducibility.

### 2.4 Statistical analysis

Continuous variables with approximately normal distributions are reported as mean +/- SD, whereas non-normally distributed variables are reported as median (interquartile range). Categorical variables are presented as number and percentage and were compared using chi-square or Fisher’s exact test, as appropriate. Group comparisons for continuous variables were performed using one-way ANOVA or Kruskal-Wallis tests according to distribution.

When a significant overall difference was found, Bonferroni-adjusted *post hoc* pairwise comparisons were performed to identify which specific groups differed from one another. Correlations between osteoactivin levels and other variables were assessed using Spearman’s rank correlation coefficient (ρ) as the data were not normally distributed. Multinomial logistic regression was then used to examine whether participants with medium or high biomarker levels had greater relative odds of belonging to the T2D or DN groups, using the lowest biomarker level group as the reference. Receiver operating characteristic (ROC) curve analysis was used to assess the diagnostic performance of circulating osteoactivin and Osteoactivin + ANGPTL8 combined in detecting diabetic nephropathy. The area under the curve (AUC), sensitivity, specificity, and the optimal cut-off value based on Youden’s Index were calculated. All statistical analyses were performed using IBM SPSS Statistics version 25.0 (IBM Corp., Armonk, NY, USA), and a two-tailed p-value of < 0.05 was considered statistically significant.

## 3 Results

### 3.1 Clinical and biochemical characteristics

**Table 1** outlines the clinical and biochemical characteristics of the three groups. Both T2D and DN groups showed significantly higher glucose and HbA1c levels compared to the non-diabetic group indicating poor glycemic control. The DN group showed the greatest degree of renal impairment demonstrated by significantly increased serum creatinine levels, blood urea nitrogen, albumin-to-creatinine ratio, microalbumin, and reduced eGFR. There were differential expression levels of circulating osteoactivin across the three groups where an increase from the control to the T2D group was observed reaching the highest levels in the DN group (P<0.001). For the angiogenic markers, Angiopoietin-2, ANGPTL4, and ANGPTL8 were significantly elevated in the DN group (P<0.05), whereas Angiopoietin-1, ANGPTL3, and ANGPTL6 showed no significant changes across the groups. Overall, the findings indicated that DN was associated with renal function decline, and higher circulating levels of both osteoactivin and angiogenic markers.

**Table 1:** Clinical and biochemical characteristics of participants. .

					Bonferroni-adjusted *post hoc* comparisons		
Variables	Non- diabetic group (N = 42)	T2DM group (N = 50)	DN group (N = 67)	ANOVA (p-value)	T2D vs. DN	T2D vs. Healthy	DN vs. Healthy
Gender (M/F) (%)	40.5/59.5	36.0/64.0	67.2/32.8	0.001^a^			
Age (Years)	54.74 ± 8.55	58.96 ± 7.22	60.09 ± 11.29	0.015*	1.000	0.104	0.014
BMI (kg/m2)	29.90 ± 4.47	33.94 ± 6.25	34.00 ± 6.88	0.001*	1.000	0.006	0.003
SBP (mmHg)	122.50 ± 14.59	135.12 ± 20.12	134.99 ± 16.72	<0.001*	1.000	0.002	0.001
DBP (mmHg)	73.76 ± 9.83	71.34 ± 12.60	70.75 ± 10.92	0.379*	1.000	0.911	0.521
Glucose (mmol/L)	5.52 ± 0.78	8.27 ± 2.54	9.61 ± 3.94	<0.001**	0.050	<0.001	<0.001
HbA1c (%)	5.66 ± 0.56	7.85 ± 1.20	8.05 ± 1.70	<0.001*	1.000	<0.001	<0.001
T chol (mmol/L)	4.79 ± 0.99	4.15 ± 0.94	4.02 ± 0.95	<0.001*	1.000	0.006	<0.001
TG (mmol/L)	1.0 (0.7-1.3)	1.2 (0.9-1.5)	1.6 (1.2-2.2)	<0.001**	0.001	0.033	<0.001
HDL-C (mmol/L)	1.44 ± 0.39	1.25 ± 0.37	1.13 ± 0.28	<0.001*	0.207	0.026	<0.001
LDL-C (mmol/L)	2.9 (2.3-3.4)	2.3 (1.7-2.6)	2.0 (1.5-2.5)	<0.001**	0.182	0.001	<0.001
VLDL (mmol/L)	0.4 (0.3-0.5)	0.5 (0.4-0.6)	0.6 (0.5-0.9)	<0.001**	0.001	0.030	<0.001
Serum Creatinine (μmol/L)	75.69 ± 18.70	79.42 ± 25.01	118.36 ± 53.78	<0.001*	<0.001	1.000	<0.001
eGFR MDRD (mL/min/1.73 m2)	81.07 ± 13.84	79.22 ± 22.32	59.70 ± 24.57	<0.001*	<0.001	1.000	<0.001
BUN (mmol/L)	4.7 (4.0-5.7)	4.7 (3.6-5.8)	6.2 (4.6-8.8)	<0.001**	<0.001	0.538	0.001
Albumin (g/L)	40.50 ± 3.37	37.94 ± 3.54	37.28 ± 3.41	<0.001*	0.927	0.002	<0.001
ACR (mg/g)	6.6 (4.3-14.2)	9.1 (6.3-14.8)	111.2 (39.7-736)	<0.001**	<0.001	0.103	<0.001
Urine Creatinine (mmol/L)	14.75 ± 7.88	11.94 ± 6.07	9.08 ± 6.33	<0.001*	0.071	0.140	<0.001
Microalbumin (mg/L)	10.7 (4.8-21.8)	11.6 (5.7-19.5)	92.8 (45.0-423.0)	<0.001**	<0.001	0.669	<0.001
Osteoactivin (ng/mL)	21.87 ± 4.88	26.00 ± 5.12	33.06 ± 10.78	<0.001*	<0.001	0.046	<0.001
Angiopoeitin-1 (ng/mL)	3.2 (1.9-6.6)	4.2 (2.3-8.3)	4.7 (3.3-10.2)	0.036**	0.216	0.206	0.010
Angiopoeitin-2 (ng/mL)	2.35 ± 1.20	3.37 ± 1.87	4.36 ± 2.70	<0.001*	0.044	0.077	<0.001
ANGPTL3 (ng/mL)	57.19 ± 39.19	53.61 ± 26.99	47.23 ± 26.80	0.230*	0.800	1.000	0.301
ANGPTL4 (ng/mL)	17.86 ± 12.69	19.82 ± 9.04	26.92 ± 12.55	<0.001*	0.004	1.000	<0.001
ANGPTL6 (ng/mL)	32.53 ± 10.98	36.43 ± 19.17	31.84 ± 15.26	0.269*	0.361	0.709	1.000
ANGPTL8 (ng/mL)	12.76 ± 5.84	23.30 ± 11.00	29.10 ± 16.20	<0.001*	0.039	<0.001	<0.001

Data presented as mean ± SD*; median (Interquartile range)** Differences among groups were assessed using one-way ANOVA* (parametric) or Kruskal–Wallis H test** (non-parametric), followed by Bonferroni-adjusted *post hoc* comparisons. ACR, urine albumin to creatinine ratio; ANGPTL, Angiopoietin; BMI, body mass index; BUN, blood urea nitrogen; DBP, diastolic blood pressure; eGFR, glomerular filtration rate; HbA1c, hemoglobin A1c; HDL, high-density lipoprotein; LDL, low-density lipoprotein; SBP, systolic blood pressure; T chol, total cholesterol; TG, triglycerides; VLDL, very low-density lipoprotein. Statistical significance is presented when p-value<0.05. ^a^Fisher-Exact Test.

### 3.2 Circulating levels of osteoactivin and selected angiogenic markers

**Figure 1:**
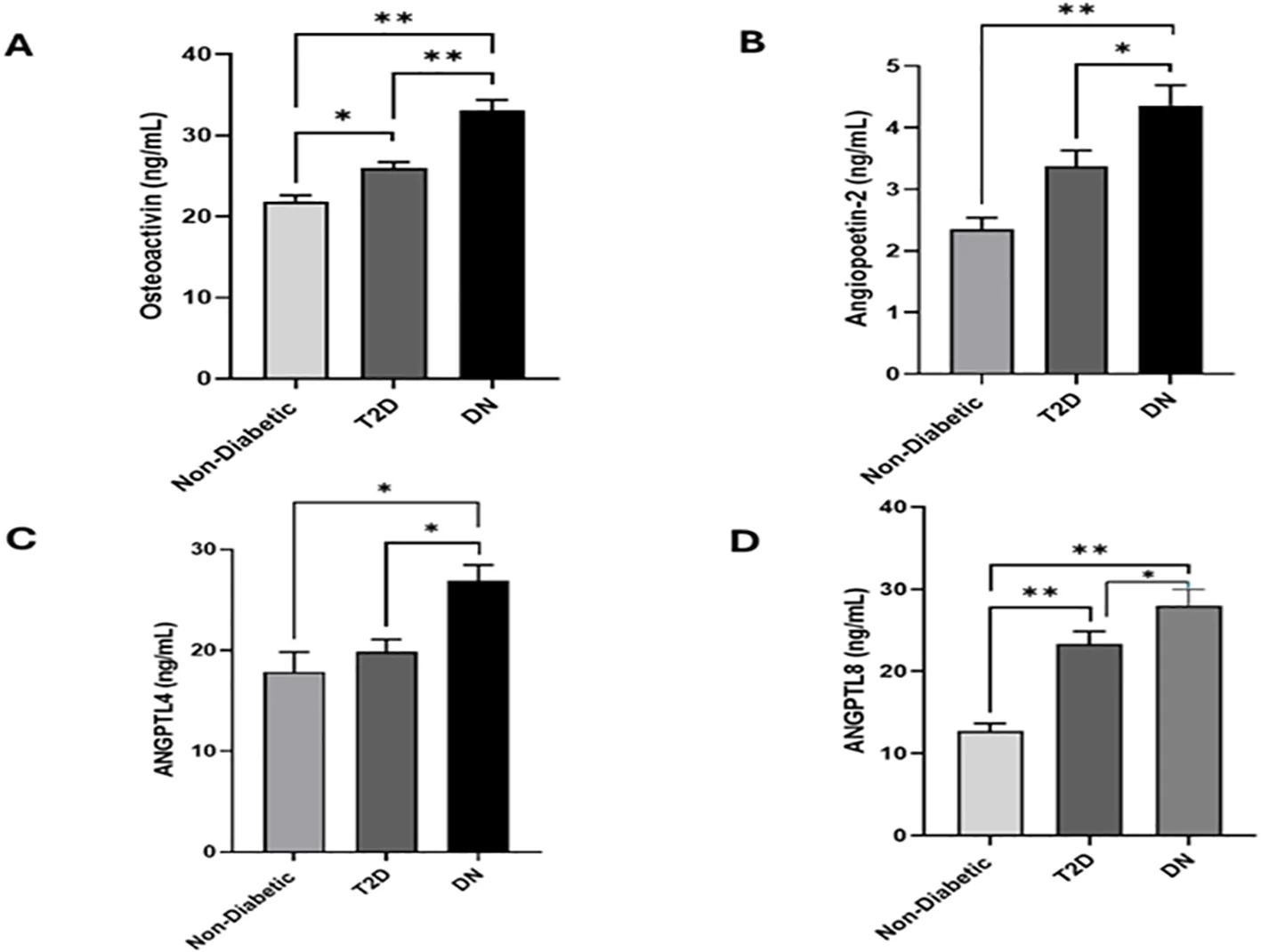
Circulating plasma levels of osteoactivin, Angiopoeitin-2, ANGPTL4, and ANGPTL8 across the three study groups; non-diabetic (n=42), T2D (n = 50), and DN (n = 67). **(A)** People with DN showed higher levels of osteoactivin compared to people with T2D. **(B)** Angiopoeitin-2 levels were higher in people with DN compared to people with T2D. **(C)** Circulating levels of IGFBP-4 were significantly higher in people with DN in comparison to people with T2D (p = 0.003). **(D)** Levels of ANGPTL8 were elevated in people with DN and the difference was significant compared to people with T2D (p < 0.01). * P < 0.05; ** P < 0.01.

**Figure 1** illustrates the differences in circulating osteoactivin and selected angiogenic markers levels across the three study groups. Osteoactivin levels were significantly higher in the T2D group compared to control, whereas the DN group demonstrated the highest levels indicating a progressive increase with disease severity (**Figure 1A**). All three of the angiogenic markers also demonstrated a similar trend where Angiopoietin-2 (**Figure 1B**), ANGPTL4 (**Figure 1C**), and ANGPTL8 (**Figure 1D**) showed significant increased levels in the DN group compared to both the control and T2D groups. Such observations indicate a possible association of the angiogenic markers with vascular dysfunction and renal injury in DN. Together, these findings suggest that both osteoactivin and angiogenic markers could potentially drive disease progression of individuals with T2D alone to develop DN.

### 3.3 Correlation of osteoactivin with renal and angiogenic markers

**Figure 2:**
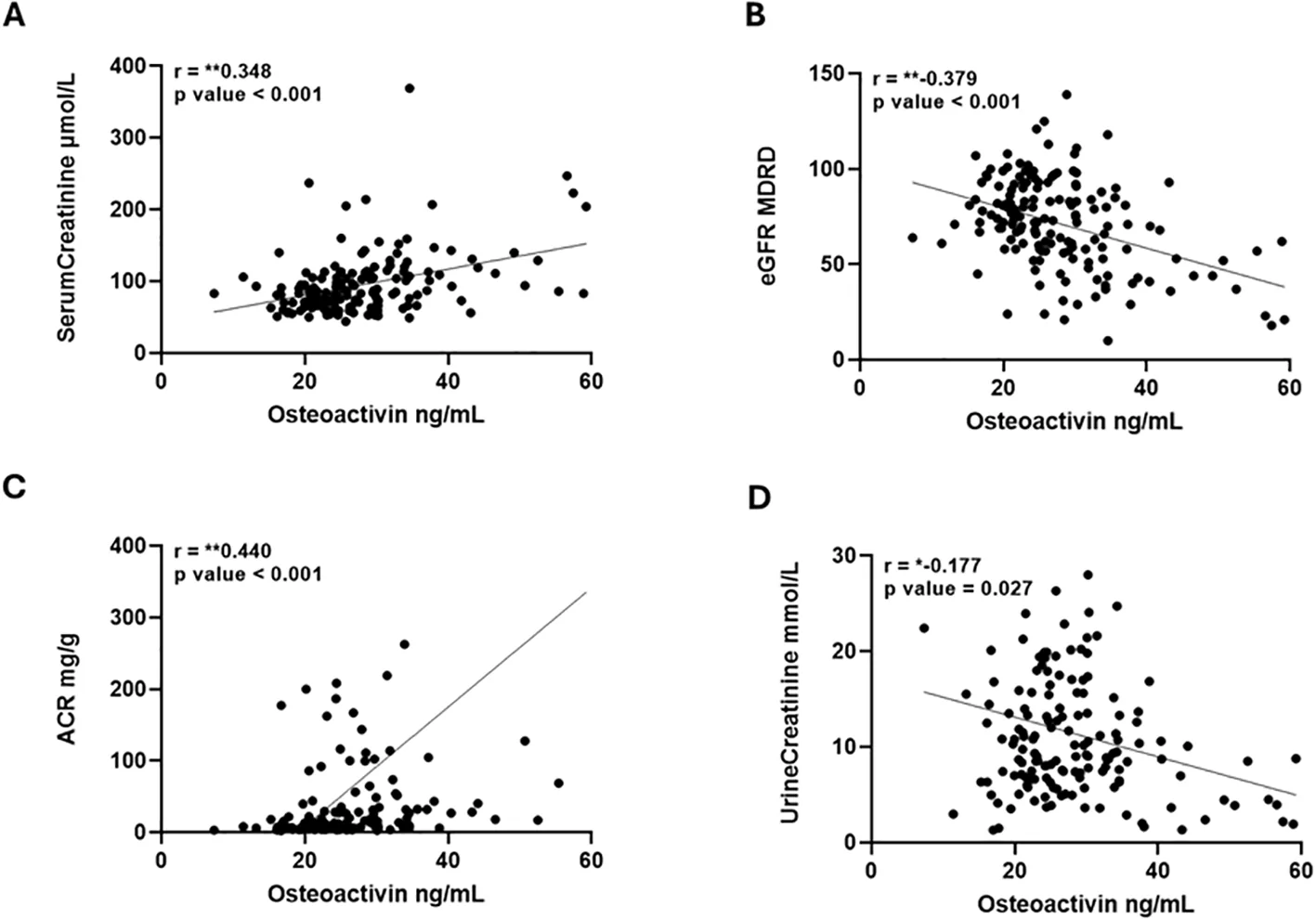
Correlation analysis between osteoactivin levels and clinical markers of kidney health. Pearson’s correlation coefficient showed a significant **(A)** positive correlation between serum creatinine and osteoactivin (r = 0.348, p < 0.001) and **(B)** negative correlation between eGFR and osteoactivin (r = -0.379, p < 0.001), and **(C)** positive correlation between ACR and osteoactivin (r = 0.440, p < 0.001) , and **(D)** negative correlation between urine creatinine and osteoactivin (r = -0.177, p = 0.59). * P < 0.05; ** P < 0.01.

Furthermore, we investigated the relationship between osteoactivin and clinical markers of kidney function. Positive correlations were observed between osteoactivin and serum creatinine (**Figure 2A**) as well as the albumin creatinine ratio (**Figure 2C**), indicating higher osteoactivin level’s potential association with markers of renal impairment. Conversely, a negative correlation was observed between osteoactivin and eGFR (**Figure 2B**) and urine creatinine (**Figure 2D**). This suggests that higher osteoactivin levels associate with a decline in overall kidney function. Although the correlation coefficients were relatively small, the findings support a consistent relationship between osteoactivin and renal dysfunction.

**Figure 3:**
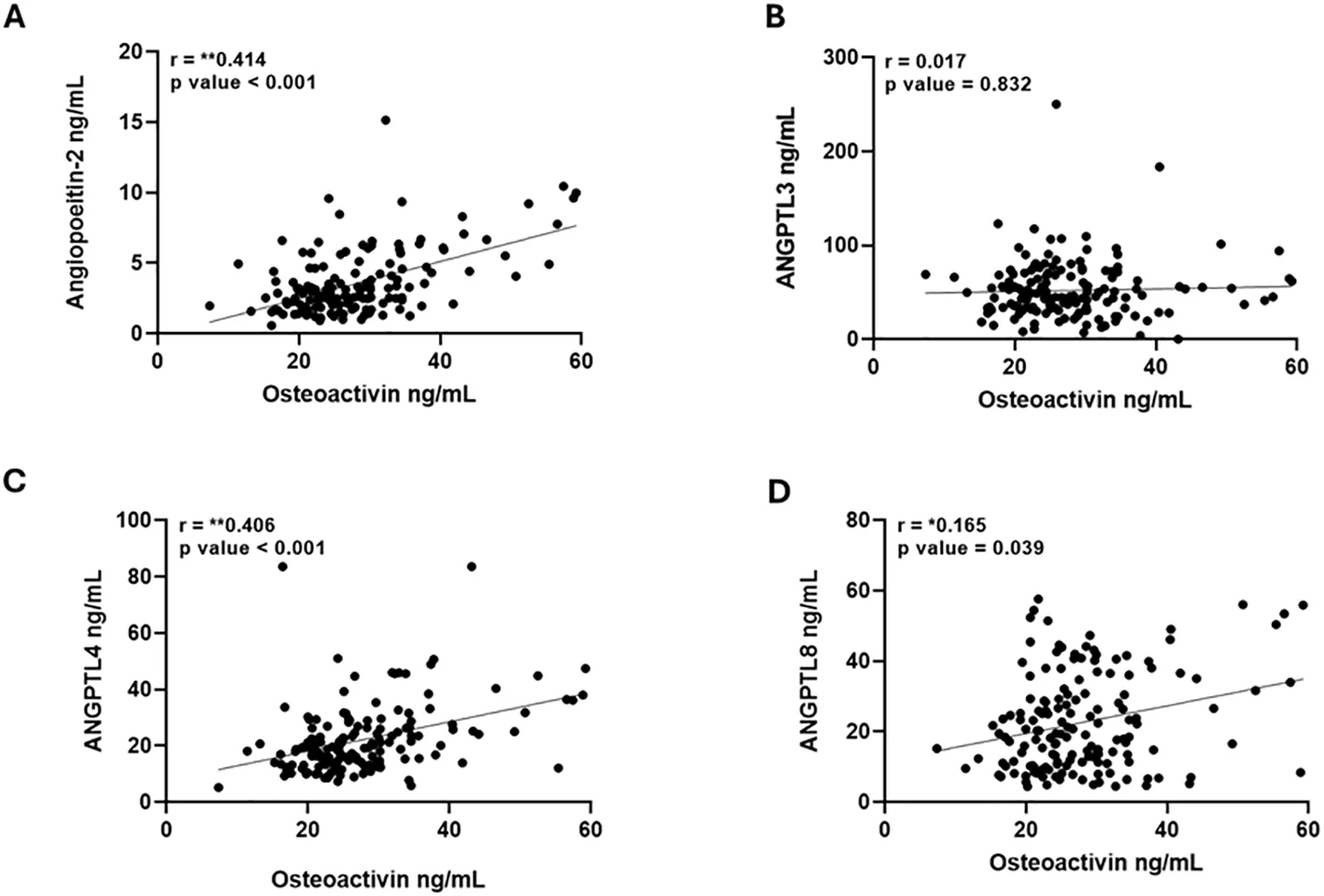
Correlations between circulating osteoactivin/GPNMB and angiogenic biomarkers, including Angiopoietin-2, ANGPTL3, ANGPTL4, and ANGPTL8. **(A)** A positive correlation was shown between osteoactivin and Angiopoetin-2 (r = 0.414, P < 0.001), (**Figure 3A**), and **(C)** ANGPTL8 (r = 0.165, P = 0.039), and **(D)** ANGPTL4 (r = 0.406, P < 0.001), and **(B)** no significant association was observed with ANGPTL3 (r = 0.017, P = 0.832). * P < 0.05; ** P < 0.01.

Similarly, we investigated whether osteoactivin levels correlate with established angiogenic markers. A positive correlation was shown between osteoactivin and Angiopoetin-2 (r = 0.414, P < 0.001) (**Figure 3A**), ANGPTL8 (r = 0.165, P = 0.039) (**Figure 3D**), and ANGPTL4 (r = 0.406, P < 0.001) (**Figure 3C**). However, no significant association was observed with ANGPTL3 (r = 0.017, P = 0.832) (**Figure 3B**).

### 3.4 Multinomial logistic regression analysis

Multinomial logistic regression analysis revealed that serum Osteoactivin and ANGPTL8 were robust, independent candidate biomarkers of both T2DM and DN when compared to healthy controls, following adjustment for age, gender, and BMI. Specifically, higher levels of Osteoactivin were significantly associated with T2DM (β=0.17, p=0.004, **Table 2**) and showed an even stronger association with DN (β=0.29, p<0.001). Similarly, ANGPTL8 demonstrated a highly significant positive relationship with both T2DM (β=0.15, p<0.001) and DN (β=0.18, p<0.001). In contrast, ANGPTL4 and Angiopoietin-2 did not achieve statistical significance in either model. Among the covariates, higher age and BMI were consistent risk factors across both groups, while male gender [or female, depending on your coding] exhibited a strong, independent negative association uniquely with DN (β=-2.73, p<0.001).

**Table 2:** Multinomial logistic regression models for T2DM (N=50) and DN (N=67) compared to healthy controls (N=42).

	T2DM vs. healthy controls (Reference)		DN vs. healthy controls (Reference)	
Predictor	Beta (SE)	p-value	Beta (SE)	P-value
Age(years)	0.08 (0.03)	**0.018**	0.08 (0.04)	**0.038**
Gender (M Vs F)	-0.99 (0.68)	0.144	-2.73 (0.78)	**<0.001**
BMI (kg/m2)	0.13 (0.06)	**0.037**	0.16 (0.07)	**0.018**
Osteoactivin (ng/mL)	0.17 (0.06)	**0.004**	0.29 (0.06)	**<0.001**
ANGPTL8 (ng/mL)	0.15 (0.04)	**<0.001**	0.18 (0.04)	**<0.001**
ANGPTL4 (ng/mL)	-0.02 (0.03)	0.528	0.02 (0.03)	0.600
Angiopoeitin-2 (ng/mL)	0.40 (0.22)	0.072	0.40 (0.24)	0.089

Healthy Controls are the reference group (N=42). SE = Standard Error. P<0.05 significant.

A multinomial logistic regression model was constructed to evaluate the predictive capacity of demographic variables (age, gender, BMI) and circulating biomarkers (Osteoactivin (ng/mL), ANGPTL8 (ng/mL), ANGPTL4 (ng/mL), and Angiopoeitin-2 (ng/mL)) in distinguishing individuals with T2DM or DN from Healthy Controls.

Values shown in bold indicate statistical significance.

### 3.5 Diagnostic performance of osteoactivin with angiogenic markers

**Figure 4:**
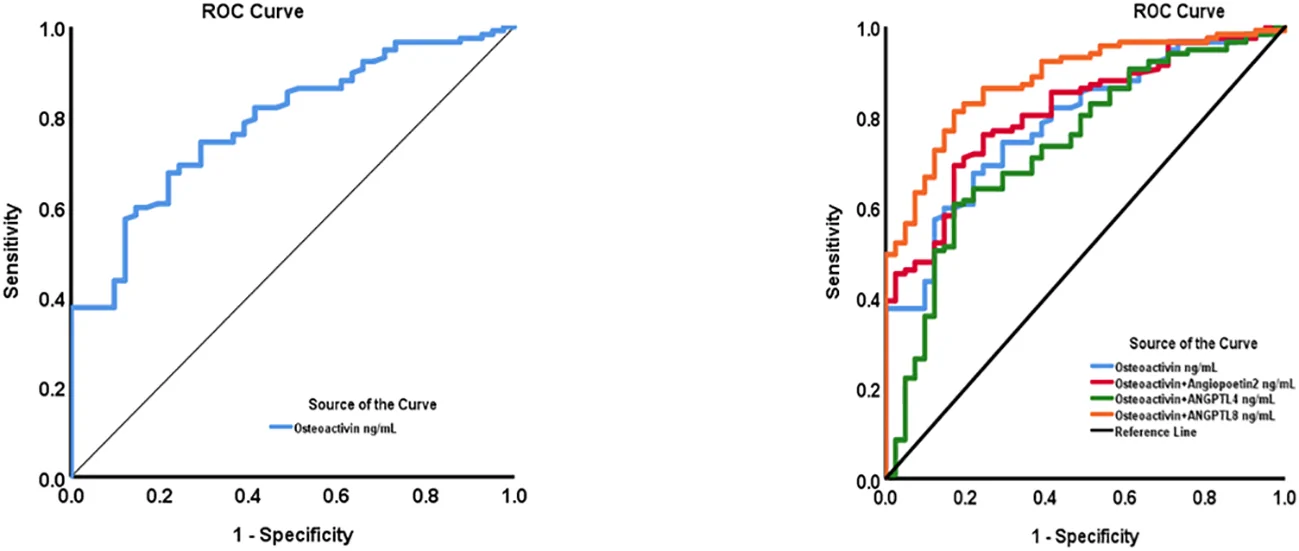
Receiver operating characteristic (ROC) curves of osteoactivin alone and in combination with angiogenic markers for the identification of diabetic nephropathy.

Receiver operating characteristic (ROC) analysis was performed to evaluate the diagnostic performance of circulating osteoactivin alone and in combination with selected angiogenic biomarkers for identifying diabetic nephropathy (DN) (**Figure 4**). Combined ROC curves were generated using predicted probabilities derived from jointly fitted binary logistic regression models, and all analyses were performed using the same listwise-complete cohort (n = 159; 117 DN/T2D cases and 42 controls). Osteoactivin alone demonstrated moderate discriminatory performance with an AUC of 0.79 (95% CI: 0.71–0.86, p < 0.001). The optimal Youden index-derived cutoff (≥24.2 ng/mL) yielded a sensitivity of 74.4% and a specificity of 68.3% (**Table 3**). The combined Osteoactivin–ANGPTL8 logistic regression model improved the diagnostic performance, achieving an AUC of 0.89 (95% CI: 0.83–0.94, p < 0.001). Using the optimal predicted probability cutoff (≥0.64), the model achieved a sensitivity of 86.3% and a specificity of 79.0%. Pairwise comparison of ROC curves was performed using the DeLong test to compare Osteoactivin alone with the combined model. Overall, these findings suggest that combining Osteoactivin with ANGPTL8 improves the discrimination of DN in this cohort; however, these findings should be considered exploratory and require validation in independent cohorts.

**Table 3:** Receiver operating characteristic (ROC) curves were performed for both Osteoactivin alone and the combination Osteoactivin-ANGPTL8 model using these saved predicted probabilities as the test variable to calculate the Area Under the Curve (AUC) with 95% confidence intervals (CI).

Model	AUC (95% CI)	p-value	Optimal Cut-off	Sensitivity (%)	Specificity (%)
Osteoactivin (ng/mL)	0.79 (0.71-0.86)	<0.001	≥24.2 ng/mL	74.4	68.3
Osteoactivin (ng/mL) + ANGPTL8 (ng/mL)	0.89 (0.83-0.94)	<0.001	≥0.64	86.3	79.0

AUC, Area Under Curve; 95% CI, Confidence Interval.

## 4 Discussion

In this study, circulating osteoactivin levels increased progressively with disease severity, with the lowest levels observed in healthy controls and the highest in patients with DN. Higher osteoactivin levels were associated with worsening renal function, as reflected by increased serum creatinine and ACR, along with lower eGFR and urine creatinine levels. Osteoactivin also showed moderate correlations with several angiogenic and metabolic markers, particularly Angiopoietin-2 and ANGPTL4. ROC analysis indicated that osteoactivin alone had moderate diagnostic performance, which robustly improved when combined with ANGPTL8 (AUC = 0.881). Taken together, these findings suggest that circulating osteoactivin may represent a promising non-invasive biomarker of renal injury and vascular dysfunction in diabetic nephropathy, with potential utility in disease detection and risk stratification.

The progressive increase in osteoactivin levels from healthy controls to patients with T2D and, ultimately, those with DN suggests a potential association between osteoactivin and the development of metabolic and renal injury. This relationship is plausible given the multifunctional role of osteoactivin as a glycoprotein involved in various cellular processes like inflammation, tissue remodeling, immune regulation, and cellular responses to metabolic stress (17–19). Consistent with this, our recent study has shown that circulating osteoactivin levels are higher in patients with diabetes, supporting its possible role in diabetes-related pathology (10). However, studies specifically examining circulating osteoactivin in diabetic nephropathy remain limited. Therefore, the present study extends the existing evidence by evaluating circulating osteoactivin in patients with established diabetic nephropathy and investigating its integration with angiogenic and kidney injury-related biomarkers. These findings further support the potential relevance of osteoactivin in diabetic renal complications.

We demonstrated the association between osteoactivin and markers of renal dysfunction, where the overall cohort was positively associated with creatinine, and ACR ratio, whereas they were negatively associated with eGFR. Similar patterns were seen in the DN group, suggesting that higher osteoactivin levels could induce kidney dysfunction and promote renal damage. These findings are in-line with other studies in which chronic hyperglycemia, oxidative stress, inflammation, and hemodynamic stress contribute to progressive glomerular and tubular injury (20). This further emphasizes the important role osteoactivin plays in inflammation and renal repair responses in DN (21). Taken together, this suggests a potential role for osteoactivin in regulating renal functions in DN.

The associations demonstrated in this study between osteoactivin and angiogenic markers further indicate a potential vascular component in the pathogenesis of DN. Endothelial dysfunction and dysregulated angiogenic signaling are well-established features of diabetic kidney disease, with Angiopoietin-2 in particular being linked to endothelial instability, albuminuria, and disease progression (22). Previous studies have shown that circulating Angiopoietin-2 levels correlate with renal dysfunction and may serve as a marker of endothelial injury in diabetic kidney disease (23, 24). Accordingly, the positive correlation between osteoactivin and Angiopoietin-2 observed in the present study, together with the elevated Angiopoietin-2 levels detected in DN patients, aligns with existing studies and suggests that osteoactivin may reflect not only renal impairment, but also the endothelial and angiogenic disturbances associated with DN. Other angiogenic and TGF-β-related proteins have also been implicated in the pathogenesis of diabetic kidney disease. Among these, leucine-rich α-2-glycoprotein 1 (LRG1) has been associated with renal dysfunction and eGFR decline in both adult and pediatric diabetic populations, reflecting its role in pathological angiogenesis and vascular remodeling (25, 26).

Similarly, both ANGPTL4 and ANGPTL8 levels were significantly elevated in the DN group and positively correlated with osteoactivin and independently associated with DN in the regression analysis. These findings are consistent with previous studies suggesting that ANGPTL4 may serve as a biomarker of diabetic kidney disease, as increased circulating levels have been associated with reduced eGFR, albuminuria, and greater disease severity (27). Likewise, ANGPTL8 levels increased progressively across the study groups and showed positive associations with osteoactivin, while also demonstrating the strongest association with disease status in the multinomial logistic regression analysis. Previous reports have similarly described elevated circulating ANGPTL8 levels in patients with diabetic nephropathy, with emerging evidence suggesting a potential role in inflammation and fibrosis within the diabetic kidney (28, 29). The enhanced diagnostic performance observed when osteoactivin was combined with ANGPTL8 may indicate that these markers reflect complementary, rather than identical, aspects of disease pathology, including metabolic stress, renal injury, inflammation, and vascular dysfunction.

The clinical relevance of osteoactivin was further supported by the multinomial logistic regression analysis presented in this study. Participants within the highest osteoactivin tertile had significantly greater odds of belonging to both the T2D and DN groups, with stronger associations observed in patients with DN. This relationship suggests that increasing osteoactivin levels may reflect increasing disease burden, and that osteoactivin may be more closely associated with nephropathy than with diabetes alone. Similar trends were observed for both Angiopoietin-2 and ANGPTL8, whereas ANGPTL4 demonstrated a more specific association with DN. Collectively, these findings suggest that osteoactivin may have greater clinical relevance when evaluated as part of a broader biomarker panel rather than as a standalone marker.

Our ROC analysis further highlighted the potential diagnostic value of osteoactivin. While osteoactivin alone demonstrated moderate discriminatory performance, its diagnostic accuracy improved when combined with angiogenic markers, particularly ANGPTL8, which yielded the highest AUC. These findings support the potential use of a multi-biomarker approach where metabolic, inflammatory, vascular, and renal injury pathways interact, which may better reflect the complex and multifactorial nature of DN. Integrating osteoactivin with other biomarkers may therefore improve diagnostic precision, and consequently, facilitates earlier identification of patients at risk of disease progression. This is in line with recent studies investigating angiopoietin proteins in T2D and DN, that have also suggested combining biomarkers as a means for enhanced disease detection (13, 30). Nevertheless, these findings were derived from a single cohort, which may limit the applicability of the results to broader populations.

This study demonstrated several strengths like the inclusion of three clinically distinct groups that enabled comparisons across the disease spectrum, ranging from non-diabetic controls to patients with T2D and DN. In addition, osteoactivin was evaluated alongside multiple angiogenic markers, providing broader insight into the vascular and renal injury pathways potentially involved in DN. Our combined analyses of correlation, multinomial logistic regression, and ROC analysis further allowed the assessment of both biological associations and diagnostic performance. Nevertheless, some limitations of our study should be considered. First, it remains unclear whether elevated osteoactivin contributes to the development of DN or simply reflects established renal injury. Second, the relatively small sample size may have limited the statistical power and broader applicability of the findings to other broader populations. Furthermore, the DN group comprised participants with established diabetic nephropathy, defined by albuminuria and reduced eGFR. Consequently, our findings may not be generalized to early or non-albuminuric diabetic kidney disease and should be interpreted within the context of established DN. Another limitation of this study is that DN was clinically defined using diabetes status, albuminuria, and reduced eGFR rather than kidney biopsy. Moreover, diabetic retinopathy and other causes of chronic kidney disease were not systematically evaluated, which should be considered when interpreting the findings. Therefore, future studies should evaluate osteoactivin in larger prospective cohorts to determine its potential value in predicting DN and progressive renal decline over time. Further mechanistic studies are also warranted to clarify whether osteoactivin plays a direct role in renal injury, endothelial dysfunction, inflammation, or tissue repair processes. In addition, it would be important to investigate whether combining osteoactivin with biomarkers such as ANGPTL8 provides incremental diagnostic or prognostic value beyond conventional renal markers in routine clinical practice.

## 5 Conclusion

In conclusion, we show circulating osteoactivin levels were elevated in individuals with T2D and were highest in patients with DN. The progressive elevation observed across disease stages, together with its strong association with angiogenic mediators, suggests that osteoactivin may reflect key pathophysiological processes underlying DN progression. Osteoactivin also demonstrated moderate diagnostic performance that improved when combined with other biomarkers such as ANGPTL8, supporting the potential value of a multi-biomarker approach in DN. Collectively, our findings suggest that osteoactivin may potentially serve as a promising biomarker for the detection and monitoring of diabetic renal complications.

## Data Availability

The original contributions presented in the study are included in the article/supplementary material. Further inquiries can be directed to the corresponding authors.

## References

[1] Thipsawat S . Early detection of diabetic nephropathy in patient with type 2 diabetes mellitus: a review of the literature. Diabetes Vasc Dis Res. (2021) 18:14791641211058856. doi: 10.1177/14791641211058856 34791910 PMC8606936

[2] Samsu N . Diabetic nephropathy: challenges in pathogenesis, diagnosis, and treatment. BioMed Res Int. (2021) 2021:1497449. doi: 10.1155/2021/1497449 34307650 PMC8285185

[3] Li X Lu L Hou W Huang T Chen X Qi J . Epigenetics in the pathogenesis of diabetic nephropathy. Acta Biochim Biophys Sin (Shanghai). (2022) 54:163–72. doi: 10.3724/abbs.2021016 35130617 PMC9909329

[4] Singh M Del Carpio-Cano F Belcher JY Crawford K Frara N Owen TA . Functional roles of osteoactivin in normal and disease processes. Crit Rev Eukaryot Gene Expr. (2010) 20:341–57. doi: 10.1615/critreveukargeneexpr.v20.i4.50 21395506

[5] Genitsaridi I Salpea P Salim A Sajjadi SF Tomic D James S . 11th edition of the IDF Diabetes Atlas: global, regional, and national diabetes prevalence estimates for 2024 and projections for 2050. Lancet Diabetes Endocrinol. (2026) 14:149–56. doi: 10.1016/s2213-8587(25)00299-2 41412135

[6] Alkandari A Alarouj M Elkum N Sharma P Devarajan S Abu-Farha M . Adult diabetes and prediabetes prevalence in Kuwait: data from the cross-sectional Kuwait Diabetes Epidemiology Program. J Clin Med. (2020) 9:16. doi: 10.3390/jcm9113420 33113867 PMC7694112

[7] Lin J Zhang P Huang Y Wei X Guo D Liu J . Elevated circulating Gpnmb levels are associated with hyperthyroidism. Endocr Connect. (2020) 9:783–92. doi: 10.1530/ec-20-0240 32688342 PMC7487193

[8] ElSayed NA Aleppo G Aroda VR Bannuru RR Brown FM Bruemmer D . 11. Chronic kidney disease and risk management: standards of care in diabetes-2023. Diabetes Care. (2023) 46:S191–202. doi: 10.2337/dc25-s011 36507634 PMC9810467

[9] McGrath K Edi R . Diabetic kidney disease: diagnosis, treatment, and prevention. Am Fam Physician. (2019) 99:751–9. 31194487

[10] Cherian P Al-Khairi I Jamal M Al-Sabah S Ali H Dsouza C . Association between factors involved in bone remodeling (osteoactivin and OPG) with plasma levels of irisin and meteorin-like protein in people with T2D and obesity. Front Endocrinol (Lausanne). (2021) 12:752892. doi: 10.3389/fendo.2021.752892 34777249 PMC8588843

[11] Hu X Zhang P Xu Z Chen H Xie X . GPNMB enhances bone regeneration by promoting angiogenesis and osteogenesis: potential role for tissue engineering bone. J Cell Biochem. (2013) 114:2729–37. doi: 10.1016/b978-1-78242-301-0.00005-7 23794283

[12] David S Kumpers P Lukasz A Fliser D Martens-Lobenhoffer J Bode-Boger SM . Circulating angiopoietin-2 levels increase with progress of chronic kidney disease. Nephrol Dial Transplant. (2010) 25:2571–6. doi: 10.1093/ndt/gfq060 20179005

[13] Tsai YC Chiu YW Tsai JC Kuo HT Lee SC Hung CC . Association of angiopoietin-2 with renal outcome in chronic kidney disease. PloS One. (2014) 9:e108862. doi: 10.1371/journal.pone.0108862 25279852 PMC4184837

[14] Tamehri Zadeh SS Toth PP Shapiro MD Surma S Banach M . ANGPTL3 vital role in different kidney diseases. Current knowledge and future perspectives. Biomed Pharmacother. (2025) 188:118189. doi: 10.1016/j.biopha.2025.118189 40412362

[15] Srivastava SPP Arora AV Hamed M Urval A Dupati A Xia S . Kidney angiopoietin-like protein 4 regulates fibrotic responses in diabetic kidney disease. Front Pharmacol. (2026) 17. doi: 10.3389/fphar.2026.1803412 42064807 PMC13124996

[16] Zou H Xu Y Meng X Li D Chen X Du T . Circulating ANGPTL8 levels and risk of kidney function decline: results from the 4C Study. Cardiovasc Diabetol. (2021) 20:127. doi: 10.1186/s12933-021-01317-3 34167540 PMC8223309

[17] Ripoll VM Irvine KM Ravasi T Sweet MJ Hume DA . Gpnmb is induced in macrophages by IFN-gamma and lipopolysaccharide and acts as a feedback regulator of proinflammatory responses. J Immunol. (2007) 178:6557–66. doi: 10.4049/jimmunol.178.10.6557 17475886

[18] Saade M Araujo de Souza G Scavone C Kinoshita PF . The role of GPNMB in inflammation. Front Immunol. (2021) 12:674739. doi: 10.3389/fimmu.2021.674739 34054862 PMC8149902

[19] Prabata A Ikeda K Rahardini EP Hirata K-I Emoto N . GPNMB plays a protective role against obesity-related metabolic disorders by reducing macrophage inflammatory capacity. J Biol Chem. (2021) 297:310. doi: 10.1016/j.jbc.2021.101232 34582891 PMC8524194

[20] Arendshorst WJ Vendrov AE Kumar N Ganesh SK Madamanchi NR . Oxidative stress in kidney injury and hypertension. Antioxid (Basel). (2024) 13:36. doi: 10.3390/antiox13121454 39765782 PMC11672783

[21] Li B Castano AP Hudson TE Nowlin BT Lin SL Bonventre JV . The melanoma-associated transmembrane glycoprotein Gpnmb controls trafficking of cellular debris for degradation and is essential for tissue repair. FASEB J. (2010) 24:4767–81. doi: 10.1096/fj.10-154757 20709912 PMC2992370

[22] Luo A-J Chang F-C Lin S-L . Exploring angiopoietin-2: clinical insights and experimental perspectives in kidney diseases. Kidney Int Rep. (2024) 9:3375–85. doi: 10.1016/j.ekir.2024.09.001 39698365 PMC11652073

[23] Szymczak A Kusztal M Gołębiowski T Letachowicz K Goździk A Kościelska-Kasprzak K . High plasma angiopoietin-2 levels predict the need to initiate dialysis within two years in patients with chronic kidney disease. Int J Mol Sci. (2023) 24:10036. doi: 10.3390/ijms241210036 37373181 PMC10298020

[24] Li M Popovic Z Chu C Reichetzeder C Pommer W Krämer BK . Impact of angiopoietin-2 on kidney diseases. Kidney Dis (Basel). (2023) 9:143–56. doi: 10.1159/000529774 38306230 PMC10826602

[25] Chen C Zhang J Yu T Feng H Liao J Jia Y . LRG1 contributes to the pathogenesis of multiple kidney diseases: a comprehensive review. Kidney Dis (Basel). (2024) 10:237–48. doi: 10.1159/000538443 38799248 PMC11126829

[26] Yang T Xu H . The critical role of LRG1 in pathological fibrosis. J Mol Med (Berl). (2025) 103:1317–32. doi: 10.1007/s00109-025-02596-z 41071357 PMC12675565

[27] Wang Y Li K Yuan S Yu C Yin R Wang D . Angiopoietin-like 4 is a potential biomarker for diabetic kidney disease in type 2 diabetes patients. J Diabetes Investig. (2024) 15:1763–72. doi: 10.1186/s12902-020-00657-7 39264678 PMC11615698

[28] Abdelnaem E Farag N Ashour R Najib A Meshref D . Role of angiopoietin like protein-8 as a predictive marker of diabetic nephropathy in type 2 diabetes mellitus. Minia J Med Res. (2025) 36:58–73. doi: 10.21608/mjmr.2025.382726.1957

[29] Pan L He Y Xiang Y Mao B Meng X Guo Y . Angiopoietin-like protein 8 mediates inflammation and fibrosis of tubular cells in diabetic kidney disease progression by interacting with Akt2. Metabolism. (2026) 174:156418. doi: 10.1016/j.metabol.2025.156418 41110501

[30] Zhao S Yang C Gao L Fan X Liu S . Unveiling the role of angiogenesis-related biomarkers in diabetic nephropathy via transcriptomics. Diabetol Metab Syndrome. (2025) 18:18. doi: 10.1186/s13098-025-02044-5 41366482 PMC12801507

